# Supplementary material for: Brief communication: Chest radiography score in young COVID-19 patients: Does one size fit all?
Source: PLoS One. 2022 Feb 23;17(2):e0264172. doi: 10.1371/journal.pone.0264172 (PMC8865641; doi:10.1371/journal.pone.0264172)
Supplement: Attachment — Submitted filename: Response to reviewer PLOS ONE.doc [file pone.0264172.s001.doc]

**Journal Requirements:**

**1. Please ensure that your manuscript meets PLOS ONE's style requirements, including those for file naming.**

*The manuscript has been amended as requested.*

**2. Please amend your Methods section to state the full name of your institutional ethics board and please provide the same information stated in your Ethics Statement regarding the waiver of consent.**

*The full name of the institutional ethics board and the waiver of consent has been added (page 3 lines 43-45)*

**Reviewers' comments:**

**Reviewer #1,** **comment 1:** The study design as mentioned lack the good number of Patients but the most important clinical error it lacks standardization as we know CXR to reveal opacities and lung shadows specially consolidation and GGO in cases of COVID 19 it should be standard.

***Response to reviewer #1, comment 1:*** *As stated also by the reviewer we mentioned that sample size is a limitation of our contribution. However, it is important to underline that all subjects were fully characterized, and consecutively collected in order to reduce selection bias. Moreover, regarding CXR standardization it’s crucial to mention the peculiar health system organization from the beginning of pandemic with a single Radiology Unit dedicated to COVID-19 patients from the Emergency Department, with the same CXR equipment, staff and setting. This point is now better clarified in the Material and Methods section (page 3, lines 49-51). Furthermore, multiple CXR scores have already been standardized as prognostic tools both in COVID-19 and ARDS, as we now included in the introduction (page 3, lines 34-38).*

**Reviewer #1, comment 2:** Exposure is one factor, type of CXR PA versus AP will affect the reading of CXR , we know also the different radiologists may read Same CXR differently and inter observer variability if a factor that limit CXR scoring , these points were not clearly delineated in the study

***Response to reviewer #1, comment 2: we agree with reviewer’s comment and*** *according to Toussie’s score method, which we aimed to validate in a different cohort, patients were included if underwent either digital anteroposterior chest radiography (47 of 51, 92%) or digital posteroanterior and lateral chest radiography (4 of 51, 8%). This point is now better explained in the Materials and Methods section (page3, lines 48, 49) and Results section. Interobserver variability was a major concern, thus readers were trained for applying Toussie’s CXR score, obtaining a moderate to substantial interobserver concordance among two different readers (Cohen's kappa coefficient (κ) ranging from 0.55 to 0.66 in different lobes).*

**Reviewer #1,** **comment 3:** There is overlap between different causes of GGO and consolidations even in COVID as pulmonary edema and pleural effusion that might be misinterpreted in CXR as consolidation, it was not clearly identified in the study.

***Response to reviewer #1, comment 3:*** *We agree with the reviewer, CXR interpretation can be confounded by comorbid conditions, like heart failure or chronic lung disease. However, considering that our patients were under 50 years old, the incidence of comorbidities that could lead to CXR misinterpretation within this age was very low (3 of 51, 6%).*

**Reviewer #2:** The manuscript must be checked by any grammar program.

Abstract

• Amend “patients’ prognosis” To “patients’ prognoses”

• Amend “In our study” To “In our study,”

Introduction

The introduction was too short and I think it will be better to conclude more studies about CXR scores and its prognostic value.

• Amend “Aim of our study was to assess the value of this” To “Our study aimed to assess the value of this”

Materials and Methods

1. Description of the methodology was concise and contained summarized description of well-established selection parameters.

2. The number of patients was suitable (51 patients) but it could be statistically accepted.

3. The choice of young and middle age was interesting.

4. Inclusion and Exclusion criteria seem logical.

5. Amend “CXR was divided in” To “CXR was divided into”

6. Amend “score equal or major to 3 as cut-off to categorize” To “score equal or major to 3 as a cut-off to categorize”

7. Amend “Clinical outcomes assessed in our cohort were: need for high-intensity” To “Clinical outcomes assessed in our cohort were: the need for high-intensity”

Results and Discussion

1. The results were represented by nice and informative radiographic images.

2. The study findings were adequately discussed.

Research criteria (1 = Excellent) (2 = Good) (3 = Fair) (4 = poor)

Originality 3

Contribution to the field 3

Technical quality 3

Clarity of Presentation 2

Depth of Research 3

Decision

The study is interesting. Therefore, I recommend it for publication after minor corrections.

***Response to reviewer #2.*** *We would like to thank the reviewer for all the comments. We amended the manuscript accordingly to your suggestions as you can see in the revised version. Moreover, in the introduction, we now mentioned a larger number of studies on CXR scores and their prognostic value (page 3 lines 34-38). We are glad that the reviewer had considered our results well discussed and our methodology suitable for this study. We are aware of the small sample size (as we stated in study limitations), however the limited number was also due to the consecutive enrollment we on purpose applied to reduce selection bias.*

**Reviewer #3:** The present brief communication provides interesting data on role chest radiography score in young COVID-19 patients. The study is well presented and proper analysed. Just a few suggestions:

1) it is known from literature that higher d-dimer level is significantly associated with a worse prognosis even in young and middle aged patients (i-e. Bonifazi et al Journal of Clinical Medicine 2021). It would be interesting, if available, to include baseline values in the multivariate analysis. If not available, i suggest to mention the lack of infomation on clinical features likely to influence outcomes, as limitation.

2) Were baseline CTs available for study population? if yes, il would be interesting to correlate CT scores with Rx scores

3) Among study limitations i would mention also the retrospective nature of the study

***Response to reviewer #3.*** *We thank the reviewer for comments and suggestions that improved our manuscript.*

1) *The cited article is very interesting and supports our results on the importance of the BMI in young COVID-19 patients, we did not quoted it before because it was not published at the time of our submission; now it is mentioned in page 6 lines 94-96. We then repeated the multivariate analysis including baseline D-dimer level, that was available in our subjects. The new analysis confirmed that high radiological risk score, diabetes and the need of high-intensity medical setting were independent risk factors for prolonged hospitalization also including baseline D-dimer, that was not a risk factor itself (OR 0.998. CI 95%: 0.990-1.005; p=0.556).*
2) *Unfortunately baseline CTs in this cohort were not available, because in our hospital during the first wave, CT scans were not largely and easily available for all patients, as it would have been later in the second wave.*
3) *Thank you for the suggestion, we included the suggested limitation in the discussion (page 7, line 103).*
