# Supplementary material for: Brief communication: Chest radiography score in young COVID-19 patients: Does one size fit all?
Source: PLoS One. 2022 Feb 23;17(2):e0264172. doi: 10.1371/journal.pone.0264172 (PMC8865641; doi:10.1371/journal.pone.0264172)
Supplement: Attachment — Submitted filename: Response to reviewer n. 2 PLOS ONE.doc [file pone.0264172.s002.doc]

**Reviewers' comments:**

**Reviewer #1, comment:** As stated by the authors the number enrolled in the study is statistically low to generate a conclusion that can be accepted for practical use , I would recommend a multi centre collection of cases specially it is a retrospective study and can easily get higher number than selected

***Response to reviewer #1, comment:*** *in full agreement with the reviewer, we’ve already emphasized that small sample size is a limitation of our contribution. However, it is important to underline that all subjects were fully characterized, and consecutively collected in order to reduce selection bias. Of note, we’ve chosen not to extend the study population to the so called “second and third pandemic wave” because several treatments have been put in place (i.e. steroid treatment) which could have influenced the outcome. Indeed we believe that “first pandemic wave” represents a unique population which is difficult to merge with the subsequent pandemics. We agree with the reviewer that multicentric studies could lead to more significant results, however this topic will be addressed together with other centers in future studies. We added the monocentricity of this study to our limitations in the Discussion section (page 7, line 114-115).*

**Reviewer #1,** **comment 1:** From symptom onset to CXR is seen shorter in mild group compared to severe group despite that it is not statistically significant but as we know covid pneumonia is a progressive disease and if the patient is presenting late , his CXR might show more shadows, which might reflect late presentation in the severe group , so I can not judge that patients with mild CXR shadows are expected to have milder form of the disease, as it will progress in the few coming days , it was not stated in the methods patients who might come twice to the hospital with worsening symptoms and condition are excluded or not, if not which CXR was selected

***Response to reviewer #1, comment 1:*** *We agree with the reviewer on this point, actually even if there was no statistical difference in the two groups, we searched for multiple access to the ER. Of note, all patients have been hospitalized after the first ER admission. Following the reviewer’s comment we clarified this point in the Materials and Methods (page 4 lines 51-52) and Results sections (page 4 lines 69-70).*

**Reviewer #1, comment 2:** As many cases of covid pneumonia are are afebrile throughout their illness , I feel fever in the table is of no value , and as oxygen saturation and respiratory rate are important vital signs in assessment of respiratory affection in covid pneumonia and it is easily assessed in all hospitals at presentation, I would recommend adding these parameters in the table beside or instead of fever

***Response to reviewer #1, comment 2:*** *We apologize, but the aim of our study was to replicate Toussie’s study on Radiology, so we kept the same parameters in our evaluation. We agree with the reviewer to add an evaluation of the gas exchanges, we used the P/F ratio, avoiding the bias of peripheral oxygen saturation due to oxygen supplementation. Confirming previous literature, P/F ratio was significantly lower (p=0.03) in HRRS patients. We then included this result in Table 1, in Results (page 6 lines 89-91) and Discussion section (page 7 lines 103-104). Unfortunately, respiratory rate was not routinely mentioned in medical records, and over 40% of the patients presented only semantic formulas like eupneic or tachipneic. Due to this lack of data we preferred not to include this parameter in our study.*

**Reviewer #1,** **comment 3:** it was not mentioned in the methods who assessed the CXR shadows, and if they were aware about the patient clinical status and if there is any bias in their interpretation of CXR reading , is there any interobserver variability

***Response to reviewer #1, comment 3:*** *We thank the reviewer for this comment which is helpful to improve our contribution.; we therefore amended Materials and Methods (page 4 lines 54-56 and lines 60-61) and Results section (page 4 lines 71-73) including the required information.*

**Reviewer #3:** I think this is a good work. the authors have properly answered to all my questions, I have no further comments

***Response to reviewer #3:*** *We thank the reviewer for the kind comment.*
